# Ovarian cancer in BRCA1 and BRCA2 gene mutation carriers: analysis of prognostic factors and survival

**DOI:** 10.3332/ecancer.2016.639

**Published:** 2016-05-03

**Authors:** Nicoletta Biglia, Paola Sgandurra, Valentina Elisabetta Bounous, Furio Maggiorotto, Eleonora Piva, Emanuele Pivetta, Riccardo Ponzone, Barbara Pasini

**Affiliations:** 1 Unit of Obstetrics and Gynaecology, Mauriziano Umberto I Hospital, Largo Turati, 62, 10128, Turin, Italy; 2 Gynecological Oncology Unit, Candiolo Cancer Institute FPO, IRCCS, Km 3,95, SP142, 10060 Candiolo (TO), Italy; 3 Obstetrics and Gynaecology I, Sant’Anna Hospital, AOU Città della Salute e della Scienza di Torino, Corso Spezia, 60, 10126, Turin, Italy; 4 AOU Città della Salute e della Scienza, Department of Medical Sciences, Corso Bramante, 88-10126, Turin, Italy; 5 AOU Città della Salute e della Scienza, SC Genetica Medica U, Via Santena, 19-10126, Turin, Italy

**Keywords:** ovarian cancer, hereditary breast and ovarian cancer syndrome, BRCA1, BRCA2, gene mutations, prognosis

## Abstract

**Objectives:**

To compare clinical–pathological characteristics and outcome between sporadic ovarian cancer and ovarian cancer in patents with hereditary breast and ovarian cancer syndrome (HBOC).

**Methods:**

Twenty-four patients with ovarian cancer treated between 2000 and 2009 who tested positive for BRCA1/2 mutation (BRCA+) and a control group of 64 age-matched patients with no family history of breast/ovarian cancer (controls) were enrolled. Clinical–pathological characteristics, surgical outcome, overall (OS), and progression-free survival (PFS) were compared between the two groups.

**Results:**

The high-grade serous histotype was more represented in BRCA+ than in controls (70.8% versus 53.1%) (p > 0.05). BRCA+ cancers were more frequently diagnosed at stage II than controls (20.83% versus 4.69%) (p = 0.024). Radical primary surgery was performed in 70% of women in both groups, with no difference in debulking results. In patients undergoing surgery after neoadjuvant chemotherapy, in all BRCA+ patients, optimal cytoreduction was achieved (versus 70% of the controls). PFS was significantly longer for BRCA+ patients compared to controls (60 months versus 22 months; p = 0.039). No significant difference was observed in OS between BRCA+ patients and controls.

**Conclusions:**

At a median follow-up time of 46 months, BRCA+ patients have a better prognosis than controls in terms of PFS. Higher chemosensitivity of BRCA+ tumours was observed.

## Introduction

The estimated lifetime risk of ovarian cancer in BRCA1 mutation carriers is 40% to 50%, among BRCA2 mutation carriers the risk is lower, ranging from 20% to 30% [1], while the lifetime risk of ovarian cancer in the general population is 1.6%.

The mean age at diagnosis of ovarian cancer in patients with germ line BRCA1 mutations is younger than for patients with sporadic cancer [2, 3].

Most ovarian cancers associated with hereditary breast and ovarian cancer syndrome (HBOC) reported in the literature are high-grade and advanced-stage serous carcinomas, whereas borderline and mucinous tumours are uncommon [4].

Ovarian cancers in patients with HBOC are associated with unfavourable pathological characteristics but with a higher chemosensitivity, [2, 5, 6, 7, 8, 9] as described in the term ‘BRCAness’ phenotype [10, 11]. Retrospective studies [5, 10, 11, 12, 13, 14] describe also a superior outcome; however, more recent data show only a short term but not a long-term survival advantage [15].

In the present study, we compare clinical–pathological characteristics and outcome, in terms of overall survival (OS) and progression-free survival (PFS), of HBOC and sporadic ovarian cancer.

## Materials and methods

### Study population

The study was carried out on 24 ovarian cancer patients who tested positive for BRCA1/2 mutation (BRCA+) and 64 age-matched patients, without any family history of breast/ovarian cancer, not tested for BRCA mutation (controls), retrieved from the database of cases operated by the same surgical team between 2000 and 2009 at our institution. Somatic BRCA mutation in the tumours was not assessed.

The patients in both groups were treated according to the same protocol (primary cytoreductive surgery followed by chemotherapy). Patients unsuitable for upfront surgery received neoadjuvant chemotherapy, including carboplatin and paclitaxel, and secondary interval surgery when feasible. Surgical stage and histological grade and cell type were categorised according to the International Federation of Gynaecology and Obstetrics (FIGO) and World Health Organisation (WHO) standards. The relevant information was obtained from pathology reports and operative notes in the medical record.

Cytoreduction was categorised as no residual disease (TR = 0), residual disease ≤1 cm (TR ≤ 1) and residual disease >1 cm (TR > 1).

Data regarding familial and personal history, BRCA status, type of surgery performed for ovarian cancer, residual disease, pathological report and follow-up status (date of the last follow-up visit, patient condition, relapse) were recorded in the database.

Clinical data, tumour characteristics, surgical treatment, PFS, and OS were compared, evaluating differences in prognosis and tumour biology between sporadic and HBOC ovarian cancer.

The median follow-up time was 46 months (interquartile range 37.5).

Patients underwent clinical follow-up examination (clinical examination and CA 125) every three months in the first two years, every six months afterwards until the fifth year after diagnosis and every year subsequently. Abdomino–pelvic computerised tomography (CT) scan was prescribed in case of symptoms, clinical findings, or CA 125 elevation.

All patients had signed a written informed consent allowing their blinded clinical data and biological material to be used for research purposes.

## Statistical analysis

Statistical analyses were performed using the SPSS 15.0 software for Windows and STATA 13.1 SE (Stata Corporation). The date of primary treatment was used as the date of diagnosis. OS was calculated according to date of death or most recent follow-up if still alive. PFS was estimated as the time from the date of primary treatment to the date of recurrence.

Two-sided *p*-value <0.05 was considered statistically significant. Normality of the variables distribution was tested by the Kolmogorov–Smirnov test. Categorical variables were compared using the Pearson’s chi square test, or Fisher’s exact test, and the analysis of variance (ANOVA), as count variables were compared using independent-samples t-test. Survival curves were estimated using the Kaplan–Meier method and compared with the log-rank test.

## Results

### Patients

Among BRCA+ patients, 19 women (79.2%) carried a BRCA1 gene mutation and five carried a mutation of the BRCA2 gene.

Median age at diagnosis was 54 years old, both for controls and for the BRCA+ group.

### Characteristics of ovarian tumours in patients with and without HBOC

The distribution of tumour characteristics in patients with and without HBOC ovarian cancer is reported in Table 1.

In the BRCA+ group, 70.8% of the tumours had high-grade serous histotype. In the control group, only 53.1% of tumours were high-grade serous (*p* > 0.05). Moreover, among BRCA+ patients, 25% of tumours were of undifferentiated histotype. In the control group, seven endometrioid, 10 undifferentiated, four clear cells, and four mucinous tumours were found.

Sporadic ovarian tumours were diagnosed at a more advanced stage (56.2% were stage III and 20.3% stage IV at diagnosis) as compared to BRCA+ cancers (45.8% stage III and 8.3% stage IV) (*p* > 0.05 for both stages). BRCA patients were significantly more frequently diagnosed at stage II than controls (20.83% versus 4.69%, *p* = 0.024).

### Surgery

Radical primary surgery was performed in 70% of women in both groups; in 30% of patients, with no difference between the two groups, a carboplatin–paclitaxel neoadjuvant chemotherapy was administered.

Optimal cytoreduction was achieved in 76% of patients with HBOC ovarian cancer and in 72% of controls (*p* > 0.05). After neoadjuvant chemotherapy, optimal cytoreduction was obtained in all BRCA+ patients, versus 70% in controls (*p* > 0.05).

Data are shown in Table 1.

### Survival

**Figure 1.**
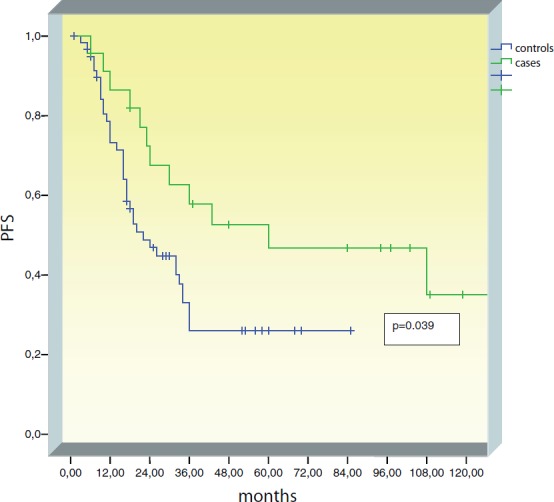
Progression-free survival (PFS) in the BRCA+ group compared to controls.

Median PFS was significantly longer in the BRCA+ group (60 months) as compared to controls (22 months) (*p* = 0.039) (Figure 1). In the control group, all recurrences occurred in the first three years of follow-up. In the BRCA+ group, recurrences have been observed up to nine years of follow-up.

No significant difference was observed in terms of OS between BRCA+ patients and controls (*p* = 0.379).

## Discussion

Ovarian cancer in BRCA mutation carriers seems to have peculiar features compared to sporadic ovarian cancer, generally showing a better outcome.

In the present study, we compared clinical–pathological characteristics and outcome of sporadic and HBOC ovarian cancer, treated at a single institution by the same team of surgeons

Ovarian cancer in HBOC is often diagnosed at a younger age compared with sporadic ovarian cancer, in particular for patients with BRCA1 mutation [1, 2, 3, 5]. Mean age at diagnosis among BRCA mutation carriers is reported to range from 45 to 48 years old in the literature [14, 16]. In our series, median age at diagnosis was 54 years old both for sporadic and for HBOC ovarian cancer.

HBOC ovarian tumours are generally diagnosed at a higher stage and grade as compared to sporadic cancers [1, 4]. On the contrary, in our study, a significant higher proportion of patients in the BRCA+ group was diagnosed at stage II when compared to the sporadic group.

Furthermore, HBOC ovarian tumours are typically of serous histology [1, 2, 16] Generally, among HBOC ovarian cancers, no borderline or mucinous cancers are reported [4]. Consistent with the literature, in the present series, most of the HBOC ovarian tumours (70%) were high-grade serous and 25% were undifferentiated tumours. On the contrary, among the sporadic tumours, only around 50% of tumours were high-grade serous and endometrioid; clear cells, and mucinous tumours were observed as well.

In our series, radical primary surgery was performed in more than 70% of patients in both groups. In one-third of the patients, both in BRCA+ and in control groups, a neoadjuvant chemotherapy was administered. In our series, in patients who underwent surgery after neoadjuvant chemotherapy, optimal cytoreduction was achieved in all BRCA+ and in 70% of controls. According to many studies, patients carrying BRCA mutation have a higher response rate to platinum-based chemotherapy [2, 5, 6, 7, 9, 10, 17]. In the study of Cass *et al*., a significantly greater response to first-line platinum-based chemotherapy and a longer disease-free interval was observed among patients with BRCA+ ovarian cancer [5]. Also, in the study of Tan *et al*., in BRCA+ patients, a significantly higher response rate to first-, second-, and third-line platinum-based chemotherapy as compared to controls was observed [10]. In the study of Gorodnova *et al*., complete clinical response was documented in 34% of BRCA mutation carriers versus 4% of non-carriers (*p* < 0.05) treated by platinum-based neoadjuvant therapy [7]. It has been suggested that the more favourable response to chemotherapy of HBOC ovarian tumours is related to their increased sensitivity to DNA-damaging agents, such as platinum-containing regimen since in the absence of BRCA1/2 function, there is a deficiency in the homologous-recombination repair, resulting in an impaired ability of tumour cells to repair platinum-induced double-strand breaks [2, 6].

Whether the superior response to platinum-based chemotherapy translates into a better outcome is unclear. Tan *et al*. described the ‘BRCAness’ phenotype, with superior outcomes following platinum-based therapy in patients with BRCA mutations [10, 18], with survival advantage demonstrated by other retrospective studies [3, 5, 8, 12, 13, 19].

A better OS in BRCA1 and 2 patients was observed by Bolton *et al*. in an analysis on 21 studies evaluating the five years overall mortality [12]. Also, the data from the Cancer Genome Atlas (TCGA) project reported a better OS in the group of BRCA mutation carriers, but only for BRCA2 carriers [13]. In the study by Gallagher *et al*. on stage III and IV ovarian cancer, a better OS but not a longer disease-free survival was observed in the BRCA+ group compared to the sporadic cancers [19]. Furthermore, Synowiec *et al*. observed a better OS in patients with BRCA1 mutations in comparison with patients with sporadic ovarian cancer, seeming BRCA1 mutation an independent prognostic factor for ovarian cancer [3]. Moreover, two recent meta-analyses show that BRCA1 and 2 carriers have a survival advantage compared to non-carriers [20, 21].

More recently, Mclaughlin *et al*. [22] Candido Dos Reis *et al*. [23] observed a short-term survival advantage for BRCA+ patients but not a long-term survival benefit. It seems that after a diagnosis of ovarian cancer, BRCA mutation confers a transient mortality benefit that diminishes with time [15]. In the recent study by Kotsopoulous *et al*., at 10 years of follow-up, 43% of non-carriers, 57% of BRCA1 mutation carriers, and 69% of BRCA2 mutation carriers died from ovarian cancer, being no residual disease at resection the strongest predictor of long-term survival [15]. According to Kotsopoulous *et al*., the initial survival advantage among women with BRCA mutations may reflect a higher initial sensitivity of BRCA carriers to chemotherapy, but this response does not predict long-term survival [15].

Previous studies found a worst outcome for BRCA+ patients, instead [24, 25]. However, their reliability has been questioned because the analysis was performed without stratifying for tumour stage.

In our study, no significant difference was observed in OS between BRCA+ patients and controls. A longer PFS was observed among BRCA+ patients as compared to patients of the sporadic group. In the control group, all recurrences occurred in the first three years of follow-up. In the BRCA+ group, recurrences occurred up to nine years after diagnosis.

## Conclusion

In our experience, BRCA+ patients have more chemosensitive tumours and longer PFS, but no OS benefit when compared to sporadic ovarian tumours.

The major limit of our study is the small sample size, while its strength is the uniformity of treatment, since all patients were treated according to the same protocol and by the same surgical team. Further multicentre studies are needed in order to obtain a larger population of carriers.

## Conflict of interest statement

The authors declare that they have no conflicts of interest.

## Figures and Tables

**Table 1.** Histological characteristics and treatment of ovarian tumours in BRCA+ patients and in controls.

		BRCA + (*N* = 24)	CONTROLS (*N* = 64)	*p* VALUE
**Histotype**	**High-grade serous**	17 (70.83%)	34 (53.12%)	>0.05^*^
	**endometrioid**	1 (4.17%)	7 (10.94%)	>0.05^°^
	**Clear cells**	0 (0%)	4 (6.25%)	–
	**Mucinous**	0 (0%)	4 (6.25%)	–
	**Undifferentiated**	6 (25%)	10 (15.62%)	>0.05^*^
	**Others**	0 (0%)	5 (7.81%)	–
**FIGO stage**	**I**	4 (16.67%)	12 (18.75%)	>0.05^*^
	**II**	5 (20.83%)	3 (4.69%)	0.024^*^
	**III**	11 (45.83%)	36 (56.25%)	>0.05^°^
	**IV**	2 (8.33%)	13 (20.31%)	>0.05^*^
	**Data not available**	2 (8.33%)	0 (0%)	–
**Primary therapy**	**Surgery**	17 (70.83%)	44 (68.75%)	>0.05^*^
	**Neoadjuvant chemotherapy (NACT)**	7 (29.16%)	20 (31.25%)	>0.05^*^
**Residual tumour after primary surgery**	**0**	13 (76.47%)	32 (72.72%)	>0.05^*^
	**≤ 1 cm**	3 (17.6%)	7 (15.9%)	>0.05^°^
	**> 1 cm**	1 (5.88%)	5 (11.36%)	>0.05^°^
**Residual tumour after surgery in NACT patients**	**0**	7 (100%)	14 (70%)	>0.05^*^
	**≤ 1 cm**	0 (0%)	3 (15%)	–
	**> 1 cm**	0 (0%)	3 (15%)	–

* tested by Fisher’s chi square exact test;

° tested by Pearson’s chi square test.
